# Prosodic discrimination skills mediate the association between musical aptitude and vocal emotion recognition ability

**DOI:** 10.1038/s41598-024-66889-y

**Published:** 2024-07-16

**Authors:** Julia Vigl, Francesca Talamini, Hannah Strauss, Marcel Zentner

**Affiliations:** Department of Psychology, University of Innsbruck, Universitätsstraße 15, 6020 Innsbruck, Austria

**Keywords:** Vocal emotion recognition, Musical aptitude, Musical expertise, Prosodic discrimination skills, Psychology, Human behaviour

## Abstract

The current study tested the hypothesis that the association between musical ability and vocal emotion recognition skills is mediated by accuracy in prosody perception. Furthermore, it was investigated whether this association is primarily related to musical expertise, operationalized by long-term engagement in musical activities, or musical aptitude, operationalized by a test of musical perceptual ability. To this end, we conducted three studies: In Study 1 (*N* = 85) and Study 2 (*N* = 93), we developed and validated a new instrument for the assessment of prosodic discrimination ability. In Study 3 (*N* = 136), we examined whether the association between musical ability and vocal emotion recognition was mediated by prosodic discrimination ability. We found evidence for a full mediation, though only in relation to musical aptitude and not in relation to musical expertise. Taken together, these findings suggest that individuals with high musical aptitude have superior prosody perception skills, which in turn contribute to their vocal emotion recognition skills. Importantly, our results suggest that these benefits are not unique to musicians, but extend to non-musicians with high musical aptitude.

## Introduction

Several studies have suggested a relationship between musical ability and the accurate recognition of vocal expressions of emotion (e.g.,^1,2^). This association has been found primarily in the context of verbal stimuli^3^, occasionally extending to tone sequences that mimic the prosody of spoken emotional expressions^4^, and to nonverbal expressions of affect, such as infant distress^5^. Conversely, amusics show impaired recognition of vocal prosody, which has been attributed to difficulties in pitch perception^6^. The prevailing research trend suggests that musical ability is specifically associated with enhanced vocal emotion recognition rather than with emotion recognition in other domains (but see^7^). This suggests that the observed benefits of musical ability are primarily due to increased auditory sensitivity, rather than advantages in cognitive abilities, such as inferring emotion from emotional cues^8^. Consistent with this view, previous research has highlighted a link between musical ability and improved perception of prosodic signals in speech^9^.

However, although the relationship between musical ability and vocal emotion recognition has been extensively studied, the factors underlying this association are not fully understood. A factor that may play a mediating role is the ability to accurately perceive subtle changes in speech prosody, as prosody is crucial for conveying emotional nuances through variations in suprasegmental melodic and temporal properties of speech phonology.

### Explaining the association between musical ability and emotion recognition skills

The domains of music and speech are closely intertwined, as evidenced by similarities in the way they are structured and how they are processed by listeners. These similarities may help explain the association found between musical ability and vocal emotion recognition (e.g.,^1,2^). Specifically, one similarity lies in hierarchical organization, whereby the higher units are defined by melodies in music and phrases in speech, while subordinate units are defined by the musical sounds and speech phonemes, both of which are processed through the same auditory pathways^10–12^. Thus, the advantage in vocal emotion recognition found among musically proficient individuals may be explained by shared neural areas and operations for syntactic processing in music and languag^13^.

Another possible explanation is that individuals with higher musical ability may have an enhanced perception of vocal modulations in speech, including those modulations that are associated with the expression of emotion^1^. This explanation is supported by research showing that, in both music and in vocal expression, emotional information is expressed through variations in characteristics such as loudness, pitch and pitch contour, timbre, tempo, rhythm, or articulation^14,15^. For example, in both domains, happiness is characterized by fast tempo and speech rate and a medium to high sound level and vocal intensity, whereas sadness is associated with slow tempo and speech rate and low sound level and intensity. Furthermore, there are parallels in pitch patterns used to express emotions, such as a descending minor third to express sadness or an ascending minor second to express anger^16,17^, which could help explain why individuals who excel at recognizing emotions in speech have also been found to have an enhanced ability to recognize emotions in music^18^. The relevant modulations in speech are often subsumed under the term prosody, which refers to suprasegmental features of the voice accompanying speech acts. These include acoustic elements (loudness, pitch), stress (intonation curves, accentuation), speaking rate, speaking style (staccato, syllables separation), and aspects of verbal planning, such as hesitation, restatement, or stuttering^19^.

The idea that prosodic discrimination skills might mediate the association between musical ability and vocal recognition of emotion is consistent with a number of findings showing that speech-related perceptual skills can be enhanced by music training. Longitudinal studies in developmental psychology indicate that children engaged in musical training experience improvements in vocal intonation and pitch perception^20^, syllable duration perception^21^, speech segmentation^22^, word discrimination^23^, and phonological awareness^24,25^. In addition, experimental studies have demonstrated that musicians have an heightened ability to detect pitch and timbre changes in both music and speech^26,27^ and advantages in speech-in-noise recognition^28^. Overall, two recent meta-analyses showed a small positive effect of musical training on auditory processing^29^, and a medium positive effect on linguistic and emotional speech prosody perception^9^. Given the importance of sensory processing in vocal emotion recognition^8,30^, these findings are particularly relevant, as evidenced by the fact that individuals with hearing loss have difficulty associating auditory cues with emotions^31^.

However, it is at present unclear whether prosodic discrimination skills mediate the relationship between musical ability and the vocal recognition of emotion. Indeed, the studies included in the meta-analysis by Jansen et al.^9^ have not treated prosodic discrimination and vocal emotion recognition as distinct components within a mediation model. In our study, we define prosodic discrimination as the detection of subtle speech variations, whereas emotion recognition focuses specifically on the identification of vocally conveyed emotions.

### Musical expertise versus aptitude

Early studies of the relationship between musical ability and vocal emotion recognition typically compared musicians and non-musicians, thus testing the effect of musical *expertise*, i.e., the effect of long-term engagement with music. However, dispositional skills in the perception of music, which are part of musical *aptitude*, do not necessarily require many years of formal musical training. Conversely, individuals with extensive musical training do not always demonstrate above-average music perception skills^32–34^. In recent years, research has increasingly moved away from the practice of contrasting musicians and non-musicians toward investigating the effect of musical aptitude as a continuous variable. For example, musical aptitude was more strongly associated with speech-in-noise perception^35^, vocal emotion recognition^36^, and according to a recent-meta-analysis, to prosody perception^9^, than musical expertise. For this reason, and to assess musical ability more comprehensibly, both musical expertise and the performance in a musical aptitude battery are included in the present studies.

### Components of musical aptitude relevant to emotional speech prosody

In striving to explain the association between musical ability and vocal emotion recognition in terms of prosodic discrimination skills, the question arises as to which components of music perception would be particularly relevant for prosodic discrimination and emotion recognition. Since not all music features have an obvious counterpart in speech prosody, we anticipate that the ability to identify emotion in vocal expressions will be associated with specific sub-abilities related to the central parameters of vocal emotion expression, rather than with general music perception skills. These parameters can be assigned to the categories pitch, loudness, temporal aspects, and voice quality^10,37,38^.

More specific acoustic cues related to these categories, according to Juslin and Scherer^37^, are components such as fundamental frequency, speech contour, and pitch jumps (which are components of pitch); speech intensity, rapidity of voice onset, and shimmer (related to loudness); speech rate, number of pauses, stressed syllables, and speech rhythm (related to temporal aspects); and high and low frequency energy in the spectrum, jitter, articulatory precision, and the slope of spectral energy (components of voice quality). Although these features do not operate in isolation, and their relevance varies from emotion to emotion, overall, the evidence described points to melody, tempo, rhythmic accents, and timbre perception as musical dimensions that should play a particularly prominent role in prosodic discrimination skills, and in turn also in the accuracy of vocal emotion recognition.

### Measurement of prosodic discrimination skills

In order to explore the potential mediating role of prosodic discrimination in the association between musical ability and vocal emotion recognition, both components need to be measured objectively and reliably. Whereas some psychometrically sound vocal emotion recognition tests exist (e.g.,^39,40^), we were unable to locate any instruments for the assessment of individual differences in prosodic discrimination ability when we started our research. Although some studies have used tasks to assess whether participants can discriminate between intonations used in statements as opposed to questions (linguistic prosody, e.g.,^41,42^), or infer emotions directly from speech samples (emotional prosody, e.g.,^43^), there appears to be a lack of psychometrically sound, construct-validated test instruments that assess prosodic discrimination skills at a lower level, namely the ability to detect subtle prosodic differences in spoken sentences. Thus, in developing the present tool for assessing accuracy in perception of subtle changes in speech prosody in Studies 1 and 2, we prioritized discriminant validity as part of construct validation, so as to ensure that the tool measures prosodic discrimination skills rather than general auditory discrimination skills. The new instrument requires participants to detect variances both in parametric manipulations, such as pitch variations, and emotional manipulations, by changing the emotional coloration through multi-parametric adjustments.

### The current studies

In the current research, we hypothesized that the association between musical ability and vocal emotion recognition would be mediated by prosodic discrimination skills. We expected that the postulated relationships would relate specifically to musical aptitude (rather than expertise), and even more specifically to the perception of those musico-acoustic parameters that are responsible for the modulation of prosodic features, namely pitch and pitch contour (i.e., melody), timbre, tempo, and rhythmical accents^10,37,38^.

In Studies 1 and 2, we created and validated a pool of stimuli designed to measure the ability to detect subtle prosodic changes in speech recordings, as the basis for a new test to assess prosodic discrimination skills. In Study 3, we tested the mediation hypothesis, and whether musical aptitude makes a stronger contribution to vocal emotion recognition than expertise. All studies were approved by the Ethics Committee of the University of Innsbruck (Certificate of good standing, 25/2022), the methods were performed in accordance with the relevant guidelines and regulations, and each participant gave informed consent prior to participation consistent with the Declaration of Helsinki.

## Study 1

The purpose of this study was to develop and validate stimuli for assessing prosodic discrimination ability. It focused on item creation, item analysis, and item reduction.

### Method

#### Participants

Eighty-five German-speaking participants (75.3% female, mean age = 24.82, *SD* = 9.39) tested the initial pool of 48 stimuli (see *Method* section). Most of participants had finished general secondary education (42.4%) or had a university degree (36.5%).

#### Stimuli to assess prosodic discrimination skills

To measure prosodic discrimination ability, we generated a task in which participants are required to determine if a test stimulus sounds same or different compared to the reference stimulus. For our initial item pool, we used 48 recordings of neutral content sentences provided by Arias et al.^44^, spoken in both neutral and emotional prosody, multiple languages and by male and female speakers. We included both neutral and emotional prosody to increase the ecological validity of the test, while also refraining from an excessive range of emotions to avoid making the test too similar to a vocal emotion recognition test.

We used 16 of these 48 items as "same" trials, meaning that they were included unchanged in our new test instrument. Half of them were recorded in neutral prosody, and the other half in emotional prosody, such as sad or happy, or fearful expressions.

To create the 32 “different” trials, we used the DAVID software^45^, which allows for precise modification of vocal signals. We modified the audio recordings in two ways. First, for 16 items, we applied parametric changes to individual or combined characteristics, such as pitch, inflection, and vibrato, without introducing emotional coloration. We achieved this by employing alternative recordings and modifying pitch and inflection. Second, for the remaining 16 items, we altered the emotional prosody by applying emotional speech transformation templates provided by the software, incorporating sound effects associated with specific emotional qualities. Thereby we changed neutral sentences to express emotions (emotional coloring), intensified already emotionally spoken sentences in their expressed emotion (emotional intensification), or changed them in their expressed emotion (emotional switch).

All audio files were exported as MP3 files and normalized to a constant volume. In total, we obtained a set of 48 stimuli, including 16 unmodified and 32 modified stimuli (see Table 1A, for an overview). Each trial consisted of three audio recordings created with Audacity^46^: First the reference stimulus, followed by its repetition 1.5 s later, and then the comparison stimulus after 2.5 s, which was either the same or different. The reference stimulus was presented twice to facilitate its encoding, thereby leaving less room for individual differences in memory capacity to affect the performance (for more detail on stimuli see Supplementary Materials, Section 1).

**Table 1:** Overview of the different items in the first and modified version of the prosody test.

A: first version before item reduction		B: second version after item reduction	
16 “same” trials	8 neutrally spoken sentences 8 emotionally spoken sentences	8 “same” trials	4 neutrally spoken sentences 4 emotionally spoken sentences
32 “different” trials	16 parametric changes: 4 alternative recordings 4 pitch modifications 4 inflection modifications 4 pitch and inflection modifications	18 “different” trials	9 parametric changes: 2 alternative recordings 2 pitch modifications 2 inflection modifications 3 pitch and inflection modifications
	16 emotional prosody changes: 4 items with emotional switch 6 emotionally colored items 6 emotionally intensified items		9 emotional prosody changes: 3 items with emotional switch 3 emotionally colored items 3 emotionally intensified items
	Total: 48 trials	Total: 26 trials	

#### Procedure

We used LimeSurvey^47^ to deliver the 48 stimuli online in a randomized order. We chose to offer five response options for each stimulus, going beyond the simple “*same*” versus “*different*” distinction to allow for more fine-grained sensory judgments^48^. First, in line with signal detection theory^49^, we included the participants' confidence level with the options “*definitely same/definitely different*” and “*probably same/probably different*”. Second, we included the additional option “*I don't know*” to avoid guessing. Participants received 1 point for each correct high confidence answer (i.e., “*definitely same/different*”) and 0.5 points for each correct low confidence answer (i.e., “*probably same/different*”). Incorrect and “*I don't know*” answers were given 0 points. The total score was calculated by summing all responses. In addition, we obtained informed consent, included demographic questions and provided a comment box for participants to give feedback on the prosodic discrimination task.

### Results and discussion

Applying general principles of item analysis^50^, we retained 26 trials that were balanced in terms of languages used, gender of speakers, parametric or emotional modifications, and difficulty, as shown in Table 1B (see Supplementary Materials, Section 2 and Table S2, for details). Of the modified stimuli, nine were edited with respect to individual or combined parameters and nine were edited with emotional speech transformations provided by the DAVID software. After item reduction, emotional prosody was restricted to happy and sad expressions.

Internal consistency of the test was *ω* = 0.84, the average total score was 17.78 (*SD* = 4.27, range = 7.5–26), and item difficulty ranged from 0.34 to 0.90 (*M* = 0.68; *SD* = 0.19). Since the test included both purely parametric and emotional components of prosody that could potentially have different mediating roles, we also examined the internal consistencies of the two subtests, which were ω = 0.71 and ω = 0.72, respectively. We created subtest scores relating to the two components and found them to be significantly intercorrelated (*r* = 0.62, *p* < 0.001). There were no significant differences in item mean scores between the four languages or between stimuli with male or female speakers, and no significant differences in total scores based on individual characteristics such as gender, education, age, or the use of headphones or loudspeakers during participation (all *ps* > 0.05).

Sensitivity was estimated using Vokey’s^51^ ${d}_{p}$, which is obtained by fitting receiver operating characteristic (ROC) curves through principal component analysis and provides a robust estimate for confidence ratings compared to traditional sensitivity measures such as ${d}{\prime}$ or ${d}_{a}$^51,52^. Average ${d}_{p}$ was 1.63 (*SD* = 0.67). The total raw scores and ${d}_{p}$ values were normally distributed, with *D*(85) = 0.09, *p* = 0.562 and *D*(85) = 0.05, *p* = 0.958, respectively. We found a strong correlation between the total raw score and the ${d}_{p}$ value (*r* = 0.94, *p* < 0.001).

In summary, Study 1 led to a novel 26-item instrument to measure prosodic discrimination ability with two subtests, that demonstrated good initial psychometric characteristics overall.

## Study 2

In this study, we examined the convergent and discriminant validity, and retest reliability of the prosodic discrimination test created in Study 1.

To establish convergent validity, we measured music perception skills using the Micro-PROMS^53^, expecting moderate correlations due to the associations between musical aptitude and auditory and linguistic perception^29^. For discriminant validity, we used two auditory tests that assess sensory thresholds for frequency discrimination and silent gap detection^54^. In accordance with general criteria for demonstrating discriminant validity^55^, we expected some association between prosodic discrimination skills and auditory thresholds, strongest for frequency discrimination, as the task primarily involved pitch modulation. Conversely, we expected a weaker correlation with the gap-in-noise task, as it does not rely on pitch discrimination.

### Method

#### Participants

A total of 93 individuals participated in the first part of the study using headphones (62% female, mean age = 23.45, *SD* = 3.53). All of these individuals completed the prosodic discrimination test and the Micro-PROMS at the initial assessment, 62 further completed both auditory tests, and 64 participated in the retest. The difference in participant numbers between the auditory tests and the PROMS can be attributed to the requirement for participants to navigate away from the primary survey via two external links for the auditory tests.

#### Measures and procedure

The study was conducted online using LimeSurvey^47^ and took approximately 30 min to complete. Two weeks after the first assessment, participants were invited to complete the prosodic discrimination task again for test–retest reliability.

For convergent validity, a novel and short version of the *Profile of Music Perception Skills*, the Micro-PROMS^53^, was employed. This test contains a total of 18 items covering all the subtests of the full version of PROMS^34^ and can be completed in 10 min. Participants were asked to indicate whether a test stimulus sounds the same or different from a reference stimulus presented twice. The items had sufficient internal consistency (*ω* = 0.64).

Two auditory tests were administered to assess discriminant validity: the *White Noise Gap Detection Test* and the *Pure Tone Frequency Discrimination Test*. These tests are classic psychoacoustic tasks that measure the sensory thresholds for frequency discrimination and silent gap detection using an adaptive procedure (e.g.,^56^) and are available on a newly developed website (http://psychoacoustics.dpg.psy.unipd.it/sito/index.php) based on the MATLAB psychoacoustic toolbox^54^. In both tests, a low threshold indicates better auditory performance. A detailed description of both tasks can be found in the Supplementary Material, Section 3.

### Results and discussion

For the prosodic discrimination test, the total score distributions, ${d}_{p}$ values and internal consistency were similar to those observed in Study 1 (see Table S2). In addition, we obtained high test–retest reliability (*r* = 0.88,* p* < *0.001*, ICC = 0.92, *n* = 64).

In terms of convergent validity, we found a moderately strong correlation between prosodic discrimination and the Micro-PROMS (*r* = 0.62, *p* < 0.001, *n* = 93). For discriminant validity, there was a moderate correlation between prosodic discrimination and the *Pure Tone Frequency Discrimination Test* (*r* = − 0.33, *p* = 0.010, *n* = 62) and a non-significant weak correlation between prosodic discrimination and the *White Noise Gap Detection Test* (*r* = − 0.10, *p* = 0.462, *n* = 62). These results suggest that, although there is some overlap between auditory perception and prosodic discrimination, they are distinct constructs, and our measure predominantly taps into the latter. Reliability, test–retest statistics and criterion correlations for the two subtests of the prosodic discrimination test were largely comparable (see Table S2).

## Study 3

In Study 3, we hypothesized that (1) both musical aptitude and expertise would be positively associated with vocal emotion recognition, with aptitude having a stronger association than expertise (e.g.,^35,36^); (2) musical aptitude would be positively associated with prosodic discrimination skills; (3) prosodic discrimination skills would be positively associated with vocal emotion recognition; and (4) the association between musical aptitude and vocal emotion recognition would be mediated by prosodic discrimination skills.

To particularize the advantage of musical ability in vocal emotion recognition (e.g.,^36^; but see also^7^), we use a multimodal emotion recognition test with auditory, visual, and audiovisual stimuli^39^. Musical aptitude was assessed using a multicomponent battery (^34^; see “Method” section), which allowed identification of the components most strongly associated with prosodic discrimination and vocal emotion recognition ability. We expected the strongest correlations with the melody, pitch, timbre, accent, and tempo subtests, based on previous literature^10,37,38^.

### Method

#### Participants

A total of 136 participants without hearing impairment took part in the study (61.8% female), with a mean age of 24.28 years (*SD* = 7.79, range = 18–66). The majority of participants had completed high school (74%) or university (15%). Sixty-eight percent of the participants lived in Austria, 16% in Germany, and 17% in Italy. About half of the participants (56.6%) identified themselves as non-musicians, while 43.4% identified themselves as musicians (37 amateur musicians, 21 (semi)-professional musicians). Just over half of the participants play at least one instrument or sing (57.4%), for a mean of 11.45 years (*SD* = 8.83, range = 1–62). Amateur musicians (*n* = 77) reported practicing 3.97 h (*SD* = 6.50) per week, while (semi)-professional musicians (*n* = 22) reported practicing 9.34 h (*SD* = 6.85). Self-reported non-musicians and musicians (i.e., amateur, semi-professional or professional) did not differ in age and educational level (all *ps* > 0.05).

#### Measures

##### Prosodic discrimination skills

Internal consistency of the test developed in Studies 1 and 2 was satisfactory (*ω*_*Total*_ = 0.82; *ω*_*Parametric*_ = 0.67; *ω*_*Emotional*_ = 0.69), mean item difficulty was appropriate (0.63), and the total scores (*M* = 16.32, *SD* = 4.25) were normally distributed according to a Kolmogorov–Smirnov test,* D*(136) = 0.06, *p* = 0.799. Sensitivity was again estimated using Vokey’s^51^ ${d}_{p}$, yielding an average value of 1.38 (*SD* = 0.66).

##### Emotion recognition ability

The *Emotion Recognition Assessment in Multiple Modalities Test* (ERAM^39^) was used to assess participants' ability to recognize emotions in audio, visual, and audiovisual presentations. The test consisted of 72 items from the GEMEP corpus, a collection of video clips of emotional expressions in pseudo-linguistic sentences^57^. In each of the three subtests (audio, visual, audiovisual), participants had to indicate which of 12 (pre)selected emotions was presented. The emotions included hot anger, anxiety, despair, disgust, panic fear, happiness, interest, irritation, pleasure, pride, relief, and sadness. In the audiovisual condition, full videos were presented, while in the audio subtest only the audio track of the videos was played and in the visual subtest the videos were shown without sound. The internal consistency of the subtests, calculated using the Kuder–Richardson Formula 20 for dichotomous data^58^, was lower than in the original study (α_total_ = 0.80 and no internal consistencies reported for the subtests^39^), with *ω*_total score_ = 0.69, *ω*_audio_ = 0.50, *ω*_visual_ = 0.28, and *ω*_audiovisual_ = 0.50 in the present study. Given the particular importance of vocal emotion recognition in this study, we examined the low reliability of the audio subtest and identified four items with very low (< 0.05) or negative item-total correlations. These four items were excluded prior to score calculation (ω = 0.54).

##### Musical aptitude

Musical aptitude was assessed using a short version of the *Profile of Music Perception Skills* (PROMS-S^59^). As in the full version, different aspects of music perception are tested with the eight subtests melody, tuning, tempo, accent, rhythm, embedded rhythm, pitch, and timbre. Participants listen to a reference stimulus twice and then decide whether a target stimulus is the same or different, with the same answer format and scoring as in the Micro-PROMS^53^. The internal consistency of the total score was *ω* = 0.87, whereas subtest scores ranged from *ω*_=_0.44 (timbre) to *ω* = 0.64 (embedded rhythm).

##### Musical expertise

Musical expertise, as a person's musical background and training, was assessed through five music-specific questions. Participants were asked about their musical self-assessment (1 = *non-musician*, 2 = *music-loving non-musician*, 3 = *amateur musician*, 4 = *semi-professional musician*, 5 = *professional musician*), whether they played an instrument or sang and, if so, for how many years, how many hours per week they practiced, and whether they had graduated from a music university or conservatory. As these questions had a high internal consistency (*ω* = 0.90), they were z-transformed and combined into one measure of musical expertise.

#### Procedure

The study was conducted online using the LimeSurvey software^47^. Participants were recruited through the university mailing list, flyers and posts on social networks of music universities and conservatories. Psychology students received course credits for their participation, and musicians were compensated with €10. After answering demographic and music-specific questions at the beginning of the study, participants completed the prosodic discrimination test and were then referred to the ERAM and PROMS-S. In total, the study took approximately 75 min to complete.

#### Data analysis and power

We calculated a mediation model using the PROCESS macro in SPSS (version 4.0^60^). Preacher and Hayes' bias-corrected nonparametric bootstrapping technique with 5000 bootstrap samples were used to estimate direct and indirect effects^61^. The web-based *Monte Carlo Power Analysis for Indirect Effects* application (https://schoemanna.shinyapps.io/mc_power_med/) was consulted to determine the required sample size for mediation assumptions^62^. Small to medium effects were expected for each mediation pathway. A minimum sample size of 133 subjects was required to achieve a power of 0.80.

## Results

### Descriptive results

Table 2 presents descriptive statistics and correlations between the variables relevant to our hypotheses. Participants were most successful at emotion recognition when the presentation was audiovisual, followed by visual and auditory presentations. Participants using headphones to participate (*n* = 96) did not differ from those using speakers (*n* = 40) on any of the test instruments used (all *ps* > 0.05). The pattern of correlations between musical aptitude, musical expertise, prosodic discrimination, and vocal emotion recognition conformed to expectations. In particular, only musical aptitude was significantly associated with vocal emotion recognition ability, whereas expertise was not. Regarding the two subtests of the prosodic discrimination test, the correlations with musical expertise, musical aptitude, and vocal emotion recognition did not differ significantly from each other, as determined by z-tests (see Table S3 for details).

**Table 2:** Descriptive statistics, zero-order correlations, and reliability statistics (McDonald’s Omega) presented in parenthesis.

Outcome	M (SD)	1	2	3	4	5	6
1. Musical expertise	0.00 (0.82)	[0.90]					
2. Musical aptitude	44.91 (8.37)	0.47**	[0.87]				
3. Prosodic discrimination	16.32 (4.25)	0.28**	0.62**	[0.82]			
4. Visual emotion recognition	62.48 (9.85)	0.20*	0.08	0.15^+^	[0.28]		
5. Audiovisual emotion recognition	71.60 (11.38)	0.16^+^	0.17^+^	0.19*	0.28**	[0.50]	
6. Vocal emotion recognition	60.96 (14.69)	0.13	0.22*	0.27**	0.43**	0.43**	[0.54]

^+^*p* < 0.10, *p < 0.05, **p < 0.01.

To particularize the unique contribution of aptitude as opposed to expertise, we conducted a subgroup analysis contrasting individuals with high and low aptitude within the low expertise group, as well as those with high and low expertise within the low aptitude group. The groups were derived by median splits. T-tests showed that among individuals with low musical expertise (*n* = 68), those with high musical aptitude (*M* = 66.25, *SD* = 11.57) were significantly better at vocal emotion recognition than those with low aptitude (*M* = 57.60, *SD* = 16.28), *t*(66) = − 2.16, *p* = 0.035, *d* = -0.57. Conversely, among those with low musical aptitude (*n* = 71), there was no significant difference between participants with low (*M* = 57.60, *SD* = 16.28) and high (*M* = 59.57, *SD* = 13.14) musical training, *t*(69) = − 0.50, *p* = 0.616, *d* = − 0.13. This analysis highlights that so-called “musical sleepers”^34^, i.e., untrained individuals with high musical aptitude, can also show advantages in emotion recognition, unlike trained individuals with low aptitude.

As shown in Table 3, we explored which specific components of musical aptitude were particularly associated with prosodic discrimination and emotion recognition. We found strong correlations between several PROMS-S subtests and prosodic discrimination, with the lowest correlation for accent and the highest for tempo. Vocal emotion recognition was found to be positively associated with the melody and timbre subtests, and marginally significant correlations were found for the rhythm, embedded rhythm, and tempo subtests. Finally, there were no correlations between visual emotion recognition and the PROMS-S subtests, while audiovisual emotion recognition was only correlated with the embedded rhythm subtest.

**Table 3:** Correlations between musical ability (expertise and aptitude) and prosodic discrimination ability and emotion recognition.

Variable	Prosodic discrimination	Vocal emotion recognition	Visual emotion recognition	Audiovisual emotion recognition
Musical expertise	0.28*	0.13	0.20*	0.16^†^
PROMS total score	0.62**	0.22**	0.08	0.17^†^
Melody	0.47**	0.23**	0.13	0.07
Rhythm	0.28**	0.14^†^	0.03	− 0.01
Embedded rhythm	0.46**	0.16^†^	0.08	0.21*
Tuning	0.41**	0.08	− 0.01	0.14
Accent	0.31**	0.06	0.02	0.12
Timbre	0.42**	0.20*	0.03	0.12
Tempo	0.54**	0.17^†^	0.12	0.14
Pitch	0.44**	0.15^†^	0.03	0.10

^†^p < 0.10, *p < 0.05, **p < 0.01.

The first three hypotheses, namely that there are correlations between musical aptitude, vocal emotion recognition ability and prosodic discrimination ability, and that the association between vocal emotion recognition and musical aptitude would be stronger than that with musical training, can therefore be confirmed.

#### Mediation analysis

**Figure 1:**
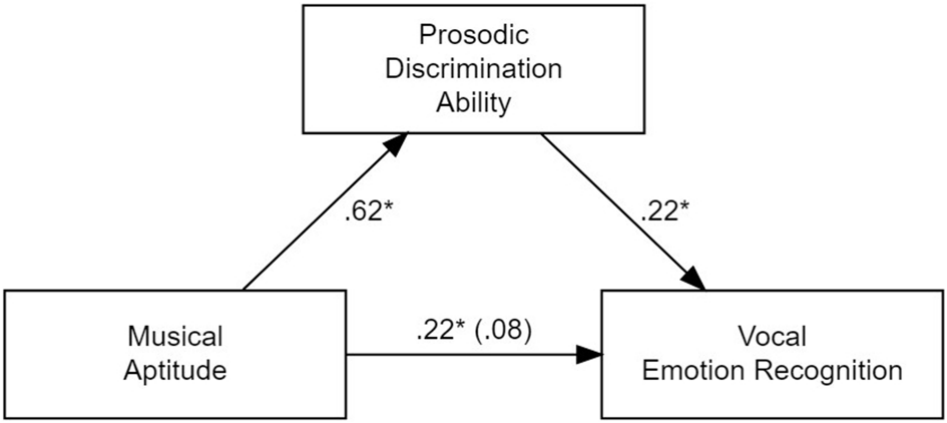
Illustration of the association between musical aptitude and vocal emotion recognition, mediated by prosodic discrimination ability. Standardized scores (β-values) are reported.

As shown in Fig. 1, we observed a significant association between musical aptitude and prosodic discrimination ability (*B* = 0.32, *SE* = 0.03, *β* = 0.62,* p* < 0.001; path A), as well as a significant association between prosodic discrimination ability and vocal emotion recognition ability (*B* = 0.76, *SE* = 0.37, *β* = 0.22,* p* = 0.040; path B). The effect of musical aptitude (*B* = 0.39, *SE* = 0.15,* β* = 0.22,* p* = 0.010; total effect C) disappeared when prosodic discrimination ability was included into the model (*B* = 0.15, *SE* = 0.19, *β* = 0.08, *p* = 0.437; direct effect C’). This result corresponds to a full mediation of the relationship between musical aptitude and vocal emotion recognition by prosodic discrimination ability (indirect effect = 0.14, 95% CI [0.01, 0.26]), thus confirming hypothesis 4.

We should note that we performed the same mediation analysis while (1) controlling for musical expertise so as to account for the possible influence of music training, and (2) excluding ERAM emotions that were present in the prosodic discrimination test (namely happiness and sadness), with no change in results.

In an additional analysis, we examined the presence of mediation for the two subcomponents of the prosody test. While it might have been expected that a mediation via the emotional test component would be more pronounced, the mediation effects of the emotional test component disappeared when the parametric test component was controlled for. More specifically, while there was a significant association between musical aptitude and prosodic discrimination skills (*B* = 0.49, *SE* = 0.07, *β* = 0.32,* p* < 0.001; path A), there was no significant association between the emotional test component and vocal emotion recognition ability (*B* = 1.19, *SE* = 0.74, *β* = 0.19,* p* = 0.110; path B) and no mediation effect (indirect effect = 0.11, 95% CI [− 0.02, 0.27]).

These results (see Table S4 for more details), underscore the essential role of both the ability to detect parametric changes and emotional changes in the voice for vocal emotion recognition.

## Discussion

In Study 3, we examined the relationships between musical aptitude, musical expertise, prosodic discrimination skills, and emotion recognition in a sample of 136 participants with varying levels of musical training. As hypothesized, our results revealed a significant association between vocal emotion recognition and musical aptitude, exceeding the strength of the association with musical expertise. The association between musical aptitude and emotion recognition was fully mediated by individuals’ prosodic discrimination skills.

### Vocal emotion recognition, musical aptitude, and musical expertise

A key finding of the present study was the robust association between vocal emotion recognition and musical aptitude, which was stronger than with musical expertise. This finding is consistent with recent research highlighting the importance of musical perceptual abilities for speech processing and vocal emotion recognition (e.g.,^9,35,36,63^), as opposed to the previous focus on musicianship (e.g.,^3^). Indeed, we found that individuals with above-average music perception abilities but no prior musical training (also referred to as “musical sleepers”^34^), showed advantages in vocal emotion recognition compared to individuals with low aptitude and training in our sample. In contrast to other studies (e.g.^3,64^), we did not find a significant association between vocal emotion recognition and musical expertise.

### The mediating role of prosodic discrimination skills

In an attempt to explain the mechanism underlying the relationship between musical ability and vocal emotion recognition, our study showed a mediating role of prosodic discrimination abilities. Consistent with the meta-analysis by Jansen et al.^9^, we observed a stronger correlation between prosodic discrimination skills and musical aptitude, compared to musical expertise. As prosodic discrimination can be distinguished from very basic perceptual abilities (Study 2), the ingredient of an advantage in vocal emotion recognition seems to lie in the enhanced perception of nuances in speech prosody that carry emotional information.

Our exploration of specific subcomponents of musical aptitude highlights the importance of melody and timbre discrimination for vocal emotion recognition, followed by rhythm, tempo, and pitch discrimination. No associations with vocal emotion recognition were observed for the tuning and accent subtests. These results are roughly in line with our expectations that perceptual abilities related to melody, pitch, timbre, tempo, and rhythmic accents should play a particular important role in vocal emotion recognition^10,37,38^.

The strong correlation between the melody subtest and vocal emotion recognition is reasonable given that emotional messages in both music and speech are conveyed through melodic patterns, such as falling pitch patterns to express sadness^10,65^. As emotion recognition in both modalities is not only based on individual sounds but rather on their progression within a musical or spoken melody, the minor role of pitch and intonation in our study is not particularly surprising. The association between vocal emotion recognition and the timbre subtest can be explained by the fact that in vocal emotion expression, timbre-like qualities such as voice tremor, shimmer, and voice roughness convey important information about emotional states^66–68^.

In contrast, musical tempo and rhythm discrimination were only marginally associated with vocal emotion recognition, while no correlation emerged for the accent subtest of the PROMS. This may be due to the different ways in which accentuation is achieved in the accent subtest compared to accentuation in speech, since the latter involves not only changes in loudness but also changes in pitch^69^.

Similarly, prosodic discrimination skills were predominantly correlated with PROMS melody perception and less with rhythm and accent perception. This seems to corroborate one finding of the meta-analysis conducted by Jansen et al.^9^ which showed that musical ability in general (expertise and aptitude) was strongly associated with prosody perception when presented in terms of pitch changes, but less so when presented in terms of timing changes. Taken together, these findings suggest that music perception in the domain of rhythm and accent may be less relevant to prosody perception and vocal emotion recognition than are skills in the area of melody and pitch perception. This interpretation does not stand in contrast to our finding that PROMS-tempo was strongly correlated with prosodic discrimination ability since, in speech, general pace can be clearly distinguished from rhythm and duration of speech elements (such as prosodic phrasing, syllable duration, e.g.,^70^).

### Direction of effects

In the present work, we tested whether prosodic discrimination skills mediate the association that has previously been found between musical ability and vocal emotion recognition. In line with prior studies (e.g.,^3,36^), we considered musical ability as an independent factor predicting speech perception and vocal emotion recognition. It should be noted, however, that the direction of effects could move in the opposite direction with advantages in vocal emotion recognition promoting musicality. To our knowledge, the literature has not yet articulated a model that moves from vocal emotion recognition to music perception skills. Although this is an interesting possibility to consider in future research, our aim here was merely to elucidate the role of prosodic discrimination skills in the musicality-to-emotion recognition association. Furthermore, even if the direction of effects were going into the opposite direction, the association between predictor and outcome would still have to be explained, and prosodic discrimination skills would again seem an obvious mediating mechanism to consider.

### Implications and future directions

The main finding of this study is that the enhanced vocal emotion recognition found in musical individuals arises from their ability to detect subtle changes in speech prosody, which is consistent with the concept of shared emotional codes across auditory channels (e.g.,^15^), musicians' advantages in speech perception^29^, and the overlapping cognitive and neural mechanisms involved in music and vocal emotion processing (e.g.,^71,72^). Although our predictions were mostly accurate, most effects were relatively small.

This may be due to the complexity of vocal emotion recognition, which involves multiple stages from sensory processing, the integration of emotionally meaningful cues, to the formation of evaluative judgments^8^. Our measures of prosodic discrimination skills and musical aptitude primarily relate to the first stage of emotion recognition, namely the perception and analysis of speech signals. On the other hand, research indicates that musical activities also affect social skills such as pro-social behavior^73^ and empathy^74,75^. Although not assessed here, these factors may influence vocal emotion recognition, especially the *interpretation* rather the *perception* stage. Our study found a modest but significant correlation between musical expertise and visual emotion recognition, suggesting potential cross-modal effects. Future research could explore the specifics of musical training, such as whether individual and group musical activities have different influences on the perception and interpretation of vocal cues.

From a research methodology perspective, our findings further highlight the limitation of inferring musical ability from musicianship status. Although musical abilities tend to be more prevalent in musically trained than in musically untrained individuals, especially those required for active music making, non-musicians can have perceptual musical skills that are on par with those of musicians. In turn, there are appreciable individual differences in music perception skills among musicians^59^ which are occluded by grouping all musicians in a single category. The noise in the data created by such classification biases may help explain the inconsistency of findings regarding the effects of musical expertise or musicianship on vocal emotion recognition (e.g.,^76,77^). As a practical recommendation for future research, we encourage the direct assessment of musical ability (e.g.,^53^).

### Strengths and limitations

The studies’ strengths lie in using comprehensive test instruments, including the development of a novel instrument for measuring prosodic discrimination ability, the integration of various subcomponents of musical aptitude into the PROMS-S, and the assessment of vocal, visual, and audiovisual emotion recognition ability using the ERAM.

A limitation is that, although several studies have shown that music perception studies conducted in the laboratory and online provide similar findings^34,78^, we cannot rule out that completing the tasks in a home environment might have introduced a certain degree of noise into the data. Another limitation is that, unlike previous studies that examined extreme groups (non-musicians vs. professional musicians), our sample included individuals ranging from non-musicians to amateur musicians, with few professional musicians. In addition, the low reliability of the ERAM may have led to some attenuation of the reported correlations^79^.

Finally, it is possible that factors not assessed in the current studies may play a role in the association between musical ability and vocal emotion recognition. One example is emotional intelligence^77^, another is personality traits, such as openness or empathy (e.g.,^80^).

## Conclusion

The present research makes two main contributions to the literature: First, it introduces a new test instrument for assessing prosodic discrimination ability; second, it sheds light on the associations between musical aptitude, musical expertise, prosodic discrimination ability, and emotion recognition ability. In Studies 1 and 2, we created a prosodic discrimination test and established its reliability and validity in assessing individuals' ability to discriminate prosodic features in vocal expressions. The mediation found in Study 3 suggests that individuals with higher musical aptitude have an enhanced ability to perceive and discriminate prosodic features that carry emotional information in vocal expressions, ultimately leading to an advantage in the recognition of emotion conveyed by the voice.

### Supplementary Information

Supplementary Information.

## Data Availability

The datasets used in all three studies, as well as the stimuli for the prosody test, are available through the Open Science Framework (https://osf.io/98f6z/).
