# Supplementary material for: Prosodic discrimination skills mediate the association between musical aptitude and vocal emotion recognition ability
Source: Sci Rep. 2024 Jul 16;14:16462. doi: 10.1038/s41598-024-66889-y (PMC11252295; doi:10.1038/s41598-024-66889-y)
Supplement: Supplementary file 1 — Supplementary Information. [file 41598_2024_66889_MOESM1_ESM.docx]

Tuning in to Emotion:

Investigating the Association between Musical Ability, Prosodic Discrimination Skills and Vocal Emotion Recognition

**Supplementary Online Material**

Content

[1. Details on Prosodic Discrimination Item Generation (Study 1) 2](#_Toc170111215)

[2. Details on Item Analysis (Study 1) 3](#_Toc170111216)

[3. Details on the Psychoacoustic Tasks Used in Study 2 5](#_Toc170111217)

[4. Additional Analyses Study 2 and 3 5](#_Toc170111218)

[References 7](#_Toc170111219)

# Details on Prosodic Discrimination Item Generation (Study 1)

To measure prosodic discrimination ability, we created a task, in which participants had to decide whether a test stimulus sounded the same or different from the reference stimulus presented to them. For the reference stimuli, we used an audio database containing recordings of six validated sentences with neutral content ^1^, performed in both neutral speech and emotional prosody ^2^. The six neutral sentences are: "I'm on my way to the meeting. ", "The airplane is almost full.", "Can you hear me?", "I'd like a new alarm clock.", "Don't forget your jacket.", and "We'll stop in a couple of minutes." In this database, the sentences are available in four languages (Japanese, English, Swedish, and French), and spoken by male and female speakers. For a first version of the prosodic discrimination task, we selected 48 audio files, 12 for each of the four languages. Six stimuli in each language were spoken by a female speaker, six by a male speaker.

We constructed a task with 16 “same” and 32 “different” trials. Half of the “same” trials were neutrally spoken sentences from the database, the other half were sentences with emotional prosody (2 sad, 3 happy, 3 fearful). We decided to modify the audio recordings of the 32 “different” trials in two ways: First, by changing individual or combined parameters (e.g., pitch, vibrato) and second, by changing the emotional prosody. We did this because there is evidence that neutral and emotional prosody are processed differently ^3^, and because previous work has examined either emotional or neutral prosody, but not both together.

To implement these modifications, we used the free software DAVID, which allows for small but precise parametric changes in the pitch, inflection, and vibrato of vocal signals, and also includes sets for emotional speech transformations that sound natural (for shading spoken sentences towards fear, joy, and sadness) ^4,5^.

For 16 items, we applied a change in single or combined parameters without emotional coloring, realized in four different ways with four trials per type:

- Alternative Recordings: In the first type, we did not artificially alter the recording, but used a second neutral recording of the same sentence by the same actor.
- Pitch modification: In the DAVID software, pitch-shift denotes the multiplication of the pitch of the original voice signal by a constant factor. In our stimuli, the pitch was increased by 50 cents (i.e., half of a semi-tone) with a delay of 10 ms.
- Inflection modification: Inflection is a rapid change in pitch at the beginning of each utterance that goes several semitones above its target, but quickly returns to normal. It is therefore perceived as more pitch variation and higher intensity. In the inflection modification of the prosody test, an inflection was inserted, with a minimum pitch change of -200 cents, a maximum change of 140 cents, and a duration of 500 ms.
- Pitch and inflection modification: In this stimuli, we combined pitch and inflection modifications. Pitch was changed by 50 cent with a delay of 10 ms. For inflection, a minimum pitch change of -400 cent and a maximum change of 50 were chosen, with a total duration of 400 ms.

For details on how each of the audio effects is implemented by the user and technically realized in the DAVID software, see ^5^ ^5^.

The remaining 16 items were edited using the emotional speech transformation templates provided by the software. These transformations are achieved by combining several sound effects and correspond to frequently identified correlates of emotional voices in the literature. For example, the transformation towards the emotion of joy involves the use of inflection, pitch increase and a filter for a brighter sound quality (see ^5^ for details).

Again, we tried to include different types of change:

- In four stimuli, we implemented an **emotional switch**. For this purpose, sad spoken sentences were processed by the software with a filter for happy prosody and vice versa.
- In six stimuli we used neutrally spoken sentences and **colored them emotionally** in the direction of sad, fearful or happy prosody.
- Finally, in six items, emotions were **amplified or attenuated**. For this purpose, emotionally spoken sentences were amplified in the same emotion, e.g., a sadly spoken sentence was additionally manipulated with an emotional transformation towards sad prosody. Similarly, for the attenuated emotions, we first amplified an emotionally spoken sentence in the same emotion and then reversed the order of presentation, i.e. the amplified stimulus was used as the reference stimulus and the original stimulus as the test stimulus.

All audio files were exported as MP3 and normalized to a constant volume. In total, we obtained a set of 48 stimuli, including 16 unmodified stimuli and 32 stimuli in two versions (see Table 1A in the manuscript, for an overview).

Each trial consisted of three audio recordings created with Audacity software: First the reference stimulus, followed by its repetition 1.5 s later, and then the comparison stimulus after 2.5 s, which was either the same or different. The reference stimulus was presented twice to facilitate its encoding, thereby leaving less room for individual differences in memory capacity to affect the performance. The same time intervals were also applied in the *Profile of Music Perception Skills* (PROMS; ^6^).

# Details on Item Analysis (Study 1)

We performed an item analysis of the trials, considering item difficulty, item-to-total-correlation, and participant feedback. No items were found to be inappropriate in terms of difficulty as defined by ^7^ ^7^, with correct responses exceeding 90% or falling below 10%. The minimum item-to-total correlation according to ^8^ ^8^ of *r* =.20 was not met by 10 items. We removed those items except one (item-to-total r =.17) that we retained to maintain balance in types of change, language, and gender of speaker. An additional 12 items were removed on the basis of participant feedback; in some cases participants noted that the changes in prosody sounded artificial, while in other cases they judged the audio quality to be substandard (e.g., background noise or static could be heard).

**Table S1**

Details on the 26 Stimuli of the Prosodic Discrimination Task after Item Reduction

| **Audio file** | **Same/ different** | **Language** | **Parametric/ emotional** | **Type of change** | **Gender of speaker** | **M** | **Item-total correlation** |
| --- | --- | --- | --- | --- | --- | --- | --- |
| EG1 | Same | Japanese | Emotional | - | Female | 0.85 | 0.27 |
| EG4 | Same | French | Emotional | - | Female | 0.79 | 0.22 |
| EG7 | Same | Japanese | Emotional | - | Male | 0.61 | 0.17 |
| EG8 | Same | French | Emotional | - | Female | 0.85 | 0.34 |
| P5G | Same | French | Parametric | - | Male | 0.70 | 0.29 |
| P6G | Same | Japanese | Parametric | - | Male | 0.64 | 0.41 |
| P7G | Same | French | Parametric | - | Female | 0.73 | 0.28 |
| P8G | Same | Swedish | Parametric | - | Male | 0.68 | 0.21 |
| ES1 | Different | English | Emotional | Neutral 🡪 sad | Female | 0.45 | 0.46 |
| ES2 | Different | Japanese | Emotional | Neutral 🡪 sad | Male | 0.62 | 0.50 |
| EH2 | Different | English | Emotional | Neutral 🡪 happy | Male | 0.62 | 0.32 |
| EHM | Different | French | Emotional | Happy 🡪 more happy | Male | 0.82 | 0.44 |
| ESW | Different | Swedish | Emotional | Sad 🡪 less sad | Male | 0.45 | 0.28 |
| EHTS1 | Different | Japanese | Emotional | Happy 🡪 more happy | Male | 0.85 | 0.56 |
| EHTS2 | Different | English | Emotional | Sad 🡪 happy | Female | 0.68 | 0.49 |
| ESTH1 | Different | Swedish | Emotional | Happy 🡪 sad | Female | 0.90 | 0.42 |
| ESTH2 | Different | French | Emotional | Sad 🡪 happy | Female | 0.90 | 0.45 |
| PS02 | Different | Japanese | Parametric | Inflection | Male | 0.86 | 0.46 |
| PS03 | Different | English | Parametric | Inflection | Female | 0.38 | 0.57 |
| PS13 | Different | Swedish | Parametric | Pitch | Male | 0.43 | 0.40 |
| PS14 | Different | French | Parametric | Pitch | Female | 0.87 | 0.37 |
| PS21 | Different | English | Parametric | Pitch and inflection | Female | 0.34 | 0.50 |
| PS23 | Different | French | Parametric | Pitch and inflection | Female | 0.53 | 0.34 |
| PS24 | Different | Swedish | Parametric | Pitch and inflection | Male | 0.44 | 0.31 |
| PS31 | Different | Japanese | Parametric | Alternative recording | Female | 0.84 | 0.25 |
| PS32 | Different | English | Parametric | Alternative recording | Male | 0.90 | 0.35 |

# Details on the Psychoacoustic Tasks Used in Study 2

The *White Noice Gap Detection Test* and the *Pure Tone Frequency Discrimination Test* administered in Study 2 are available on a newly developed website based on the MATLAB psychoacoustic toolbox ^9,10^. The chosen tests were customized for the present study to be a three-alternative forced choice tasks. Specifically, the participant had to detect which one of three tones was different (in case of the frequency discrimination) or contained a silent gap (in case of the gap detection), and the difficulty of the task was increasing/decreasing based on the participant’s correct or incorrect answer. The difference between the standard and the comparison was decreasing by a factor of two for the first two reversals, and by a factor of 1.41 for other six reversals until the task stopped. Each task was repeated twice, and the threshold was computed by averaging the last six reversals of the two repetitions. The white noise Gap Detection Test had a standard tone of white noise presented for 500ms, and the comparison stimulus had the same total duration but included an initial silent gap of 20ms. The Pure Tones Frequency Discrimination Task had a standard pure tone of 1000Hz, and the comparison was initially 100Hz lower than the standard. Each tone was presented for 500ms.

# Additional Analyses Study 2 and 3

**Table S2**

Reliability and Validity Statistics of the two Subtests of the Prosodic Discrimination Test.

|  |  |  |  |  |  | **Correlations** |  |
| --- | --- | --- | --- | --- | --- | --- | --- |
| **Subtest** | **M (SD)**  **at first measurement** | **n** | **Omega** | **Test-Retest** | *Micro-PROMS* | *Pure Tone Frequency Discrimination* | *White Noise Gap Detection Test* |
| Full prosody test | 16.16 (4.43) | 136; 64 | .83; .87 | .88** | .62** | -.33** | -.10 |
| Parametric transformations | 7.70 (2.57) | 136; 64 | .74; .78 | .80** | .51** | -.14 | -.010 |
| Emotional transformations | 8.46 (2.29) | 136; 64 | .75; .76 | .80** | .47** | -.20** | -.002 |

*Note. **p < .01. N and omegas are reported for both the test and the retest assessment.*

**Table S3**

Correlations of the two Subtests of the Prosodic Discrimination Test With Musical Ability and Vocal Emotion Recognition

|  | **Expertise** | **Aptitude** | **Vocal emotion rec.** |
| --- | --- | --- | --- |
| Prosodic Discrimination total | .28^**^ | .62^**^ | .27^**^ |
| Parametric transformations | .27^**^ | .55^**^ | .22^**^ |
| Emotional transformations | .24^**^ | .59^**^ | .27^**^ |
| z-Test | *r* = .36, *p* = .358 | *r* = -.74, *p* = .229 | *r* = -.74, *p* = .230 |

*Note. * p < .05, **p < .01.*

**Table S4**

*Association Between Musical Aptitude and Vocal Emotion Recognition, Mediated by Prosodic Discrimination Ability, Subtest Emotional Modifications, While Controlling for the Effect of the Subtest Parametric Modifications.*

| Path | *B* | *SE* | 95% *CI* | *p* | β |
| --- | --- | --- | --- | --- | --- |
| X 🡪 M (path A) | 0.49 | 0.07 | [0.34, 0.64] | < .001 | 0.32 |
| M 🡪 Y (path B) | 1.19 | 0.74 | [-0.28, 2.66] | .110 | 0.19 |
| X 🡪 Y (path C) | 0.25 | 0.18 | [-0.10, 0.59] | .163 | 0.14 |
| X 🡪 M 🡪 Y (path C’) | 0.14 | 0.19 | [-0.23, 0.51] | .465 | 0.08 |
| Indirect effect | 0.11 | 0.07 | [-0.02, 0.27] |  | 0.06 |

*Note. X = musical aptitude, Y = vocal emotion recognition, M = prosody subtest emotional modification.*

References

1. Russ, J. B., Gur, R. C. & Bilker, W. B. Validation of affective and neutral sentence content for prosodic testing. *Behav. Res. Methods* **40,** 935–939; 10.3758/BRM.40.4.935 (2008).

2. Arias, P., Rachman, L., Lind, A. & Aucouturier, J. J. IRCAM Neutral-content Voices (audio corpus). Available at https://archive.org/details/NeutralContentSaidEmotionallyDataBaseUploadedVersion (2015).

3. Pinheiro, A. P. *et al.* Abnormalities in the processing of emotional prosody from single words in schizophrenia. *Schizophrenia Research* **152,** 235–241; 10.1016/j.schres.2013.10.042 (2014).

4. Aucouturier, J.-J. *et al.* Covert digital manipulation of vocal emotion alter speakers' emotional states in a congruent direction. *Proceedings of the National Academy of Sciences* **113,** 948–953; 10.1073/pnas.1506552113 (2016).

5. Rachman, L. *et al.* DAVID: An open-source platform for real-time transformation of infra-segmental emotional cues in running speech. *Behav. Res. Methods* **50,** 323–343; 10.3758/s13428-017-0873-y (2018).

6. Law, L. N. C. & Zentner, M. R. Assessing musical abilities objectively: construction and validation of the profile of music perception skills. *PLoS One* **7,** e52508; 10.1371/journal.pone.0052508 (2012).

7. Thissen, D. & Wainer, H. *Test scoring* (Lawrence Erlbaum Associates, 2001).

8. Crocker, L. & Algina, J. *Introduction to classical and modern test theory* (Holt, Rinehart and Winstron, 1986).

9. Grassi, M. & Soranzo, A. MLP: a MATLAB toolbox for rapid and reliable auditory threshold estimation. *Behav. Res. Methods* **41,** 20–28; 10.3758/BRM.41.1.20 (2009).

10. Soranzo, A. & Grassi, M. PSYCHOACOUSTICS: a comprehensive MATLAB toolbox for auditory testing. *Front. Psychol.* **5,** 712; 10.3389/fpsyg.2014.00712 (2014).
